# *Mycoplasma pneumoniae *and/or *Chlamydophila pneumoniae *inoculation causing different aggravations in cholesterol-induced atherosclerosis in apoE KO male mice

**DOI:** 10.1186/1471-2180-9-194

**Published:** 2009-09-10

**Authors:** Sueli B Damy, Maria L Higuchi, Jorge Timenetsky, Márcia M Reis, Suely P Palomino, Renata N Ikegami, Fabiana P Santos, Junko T Osaka, Luiz P Figueiredo

**Affiliations:** 1 Medical School, University of Sao Paulo/LIM 26, Sao Paulo, Brazil; 2 Heart Institute (InCor) of Clinical Hospital, University of Sao Paulo, Sao Paulo, Brazil; 3 Institute of Biomedical Sciences, University of Sao Paulo, Sao Paulo, Brazil; 4 Adolfo Lutz Institute, Sao Paulo, Brazil

## Abstract

**Background:**

*Chamydophila pneumoniae *(CP) and/or *Mycoplasma pneumoniae *(MP) are two bacteria detected in vulnerable atheromas. In this study we aimed to analyze whether CP and/or MP aggravates atherosclerosis induced by cholesterol-enriched diet in C57BL/6 apoE KO male mice. Thirty male apoE KO mice aged eight weeks fed by a diet containing 1% cholesterol until 32 weeks of age were divided into four groups: the first was inoculated with CP (n = 7), the second with MP (n = 12), the third with both CP + MP (n = 5), and the fourth with saline (sham n = 6). The animals were re-inoculated at 36 weeks of age, and sacrificed at 40 weeks of age. Two ascending aorta and one aortic arch segments were sampled. In the most severely obstructed segment, vessel diameter, plaque height, percentage of luminal obstruction and the degree of adventitial inflammation were analyzed. The plaque area/intimal surface ratio was obtained by measuring all three segments. The adventitial inflammation was semiquantified (0 absent, 1 mild, 2 moderate, and 3 diffuse).

**Results:**

The mean and standard deviation of plaque height, % luminal obstruction, external diameter, the plaque area/intimal surface ratio and the adventitial inflammation values are the following for each group: MP (0.20 +/- 0.12 mm, 69 +/- 26%, 0.38 +/- 0.11 mm, 0.04 +/- 0.04 and 0.22 +/- 0.67), CP (0.23 +/- 0.08 mm, 90 +/- 26%, 0.37 +/- 0.08 mm, 0.04 +/- 0.03, and 0.44 +/- 0.53), MP + CP (18 +/- 0.08 mm, 84 +/- 4.0%, 0.35 +/- 0.25 mm, 0.03 +/- 0.03 and 1.33 +/- 0.82) and sham (0.08 +/- 0.09 mm, 42 +/- 46%, 0.30 +/- 0.10 mm, 0.02 +/- 0.03 and 0.71 ± 0.76). A wider area of plaque/intimal surface was observed in MP + CP inoculated groups (p = 0.07 and 0.06) as well as an increased plaque height in CP (p = 0.01) in comparison with sham group. There was also an increased luminal obstruction (p = 0.047) in CP inoculated group in comparison to sham group. Adventitial inflammation in MP + CP inoculated group was higher than MP, CP and the sham groups (p = 0.02).

**Conclusion:**

Inoculation of CP, MP or both agents in C57BL/6 apoE KO male mice caused aggravation of experimental atherosclerosis induced by cholesterol-enriched diet, with distinct characteristics. CP inoculation increased the plaque height with positive vessel remodeling and co-inoculation of MP + CP caused the highest adventitial inflammation measures.

## Background

Atherosclerosis is considered an arterial inflammatory disease resulting from lipid entrance in the vascular wall and subsequent oxidation. Lipid oxidation has been related to infectious agents [1], mainly *Chlamydophila *or *Chlamydia pneumoniae *(CP) [2-4]. CP induced or accelerated atherosclerosis in experimental animals [5-7]. Although more than 700 studies have been published focusing CP in atherosclerosis, the inconsistent results of clinical trials using antibiotic therapy discouraged the infection theory. However, our previous studies have shown that co-infection of CP and *Mycoplasma pneumoniae *(MP) is usually present in atherosclerotic plaques, in greater amount in ruptured plaques [8,9]. The co-infection theory is corroborated by the recent finding of increased serum antibodies to MP and CP in patients with atherosclerosis and acute myocardial infarction [10,11]. Fibrous cap stabilizes human atherosclerotic plaques and we found that plaque fibrosis is related to increased growth factors and higher proportion of MP to CP [12]. On the other hand, predominance of CP in such co-infection is related to plaque rupture. *Mycoplasma *is the smallest self-replicating microorganism having particular characteristics as cholesterol requirement for growth, drawing the host for immune depression [13] and increase the pathogenicity of co-infective agents [14]. Association of different microorganisms in a host may increase the virulence among them [15,16] and may explain the disappointing clinical trial results with anti-chlamydial antibiotic therapy [17,18].

The objective of the present study was to verify whether inoculation of MP or in association with CP aggravates cholesterol-induced atherosclerosis in apoE KO mice. The severity of atherosclerosis was evaluated by measuring the plaque height, plaque fat area, intima and adventitia inflammation and amount of plaque/surface of the vessel. We also evaluated whether co-infection would cause plaque rupture.

## Results

**Figure 1:**
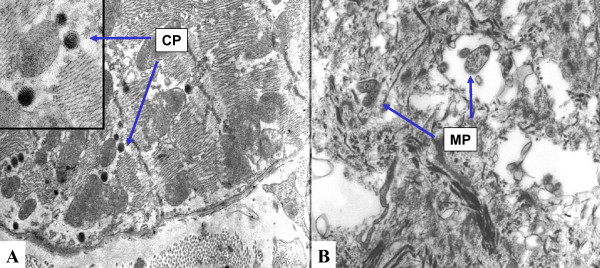
**Electron microscopic views of *Mycoplasma pneumoniae *(MP) and *Chlamydia pneumoniae *(CP) bodies**. Elementary bodies in the myocardial fiber from a mouse of the MP + CP infected group. The close view on the left side shows the double membrane of CP elementary bodies (1A). An intimal plaque from a mouse of the MP infected group, exhibiting two rounded mycoplasma bodies, characterized by only one envelopment membrane (1B).

The experimental infection caused six deaths in the 36 studied male mice: Among the death mice, four were inoculated with MP, one was inoculated with CP + MP and one was from the sham group. By the end of the experiment, the pooled serum were tested for total cholesterol, HDL and LDL in all groups. The respective values were: 534, 350, 443 and 532; HDL 29, 20, 40, 21 and LDL 435, 215, 316 and 393 mg/dl. After 4 weeks the inoculated mice showed serum antibody titers of: < 1:16 to CP, from 1:8 to 1:16 to MP and the sham did not show antibodies to CP and MP. Electron microscopic of the intimal plaque of a mouse inoculated with MP showed structures suggestive of MP such as irregular rounded bodies with 0.1 to 0.4 μm in diameter, lack of the cell wall, containing granular chromatin-like material (Figure 1). One animal of the CP + MP inoculated group exhibited the structures of MP and the elementary bodies of CP in the myocardial fiber characterized by rounded electron-dense bodies enveloped by two membranes (Figure 1A and 1B).

### Analysis of the extent and degree of atherosclerosis

Table 1 shows the mean and standard deviation of variables in the different groups. P value is the comparison of the infected groups with the sham group, using One Way Analysis of Variance and Dunn's Methods for non-normally distributed values or Bonferroni's test for normally distributed values. Two significant differences was observed: higher % of luminal obstruction in CP group compared with sham group and higher adventitial inflammation in the group co-infected with MP + CP compared with infected only with MP.

**Table 1:** Mean (SD) of variables in the cross-sections of aortic segments from infected and sham inoculated apoE KO mice

Group	Plaque/internal surface Mean (SD)	External Diameter Mean (SD)	% obstruction Mean (SD)	plaque height	Inflammation adventitia (0 - 3+)
**MP (n = 12)**	0.038 (0.037)	0.38 (0.11)	69 (26)	0.20 (0.12)	0.22 (0.67)
**CP (n = 7)**	0.043 (0.028)	0.37 (0.11)	90(26)	0.23 (0.08)	0.44 (0.53)
**MPCP (n = 5)**	0.032 (0.027)	0.30 (0.11)	84 (4.0)	0.18 (0.08)	1.33 (0.82)
**Sham inoculated (n = 6)**	0.02 (0.03)	0.30 (0.11)	42 (46)	0.08 (0.09)	0.71 (0.76)
**P **(ANOVA and Dunn's test)	0.20	0.27	0.047 (CP vs Sham)	0.07	0.02 (MP vs MPCP)
**P (T test)**	0.07 (MP vs Sham) 0.06 (CP vs Sham)			0.012 (CP vs Sham)	

MP - *Mycoplasma pneumoniae*, CP - *Chlamydia pneumoniae*, SD -Standard Deviation. P values correspond to ANOVA test and Dunn's, for non-normally distributed values or Bonferroni's test for normally distributed values.

In variables showing a trend to be different when comparing simultaneously the 4 groups, Student T test was used to compare the two groups with the highest difference. It showed significant major plaque high in CP group than the sham and a trend to have major plaque area/internal surface in MP and CP groups than in sham group.

External diameter, which indicates vessel remodeling, did not differ between infected versus sham groups. However, the animals infected with CP or MP inoculums exhibited more atheroma plaques on the intima surface (0.043 +/- 0.028 and 0.038 +/- 0.037 mm^2^/mm) than the sham group (0.020 +/- 0.03 mm^2^/mm) with no statistical significant difference (p = 0.06 and p = 0.07, respectively). The most severely obstructed atherosclerotic sites had increased plaque height in the CP group compared with sham and more adventitial inflammation in MP+CP group, compared with MP group. There was not ruptured plaque in any of the groups.

## Discussion

The present study showed that intraperitoneal inoculation of MP, CP or the both microbes aggravated atherosclerosis induced by cholesterol-enriched-diet in apoE KO male mice, as measured by plaque height, % luminal obstruction, adventitial inflammation and amount of plaque area/internal surface. This study analyzed the ascending aorta and aortic arch, which are segments of aorta that are more prone to development of atherosclerosis [5]. CP infection is associated with increased lymphocytic inflammation [9]. Particular characteristics of mycoplasma might contribute to different atheroma plaque outcomes: Mycoplasma growth depends of cholesterol viability, this microorganism has surface compounds that modulates the host immune response, cause immunosuppression and facilitates the proliferation of other infectious agents [19]. However MP seems to inhibit CP growth [11].

**Figure 2:**
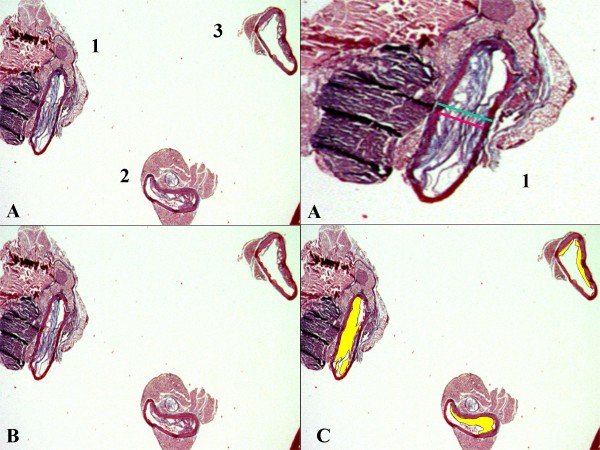
**An example of three aorta cross-sections, and how the measures were taken**. Three sections and the close view of section n°.1 corresponds to the most severely obstructed segment, where measurement of plaque height (red line) and external diameter (green line) were performed (2A). The internal vessel perimeter measurement, represented by red dotted lines (2B) and the total plaque area, in yellow (2C).

Infection by CP and co-infection by MP + CP caused more severe % luminal obstruction and adventitial inflammation than sham group or inoculation with only MP in apoE KO mice fed cholesterol-enriched-diet. The severity of % of luminal obstruction is a combination of plaque height and vessel diameter. In CP group the plaque height is high but probably associated with positive remodeling as the external vessel diameter is larger than the sham group. The MP + CP group presented smaller plaques but without vessel remodeling, the external vessel diameter presenting the same values than the sham group. The hypothesis of a flattened lumen vessel due to a lack of fixation of the vessel wall should be considered. The plaques in MP group were also associated with positive vessel remodeling. The lack of statistical significant difference in the external vessel diameter that represents the degree of vessel remodeling may be related with three factors: a) large standard deviation values and b) the site chosen for doing the measures: as exemplified in methods with the Figure 2, section 3, it was not used the plaque height but the lowest lumen value for choosing the site to be measured and c) some segments might be partially collapsed due to a lack of perfusion fixation.

The interrelationship between these microbes and different atheroma plaque morphology have already been found in human plaques. Advanced coronary atheroma plaques in humans showed that few CP and MP antigens were detected in small and fibrotic plaques, which were associated with negative vessel remodeling causing severe obstruction, and on the contrary, vulnerable plaques were rich in MP, increased adventitial inflammation that correlated with the numbers of cells positive for CP [9]. Also, in initial human atherosclerotic lesions, high MP/CP ratios were associated with increased levels of growth factors and fibrosis and low number of macrophages [12].

Similarly, in the present study, inoculation of CP was associated with increased plaque size, higher mean external vessel diameter, which are characteristics of plaque vulnerability as described in humans [9]. Favoring the co-infectious theory, human clinical studies demonstrated association of increased MP and CP antibody titers with acute myocardial infarction patients [10,11,20].

Previous studies in the literature did not show aggravation of atherosclerosis by intranasal CP inoculation in apoE KO mice in a short follow-up period [5]. Intranasal *Mycoplasma pneumoniae *inoculation in rabbits did not induce atherosclerosis in a short follow-up period [21]. In comparison to presented findings, it is believed that differences in the route of inoculation of infectious agents (intraperitoneal versus intranasal) and time of follow up may explain these apparently conflicting results. It seems that intraperitoneal inoculation is more efficient in disseminating the infectious agents than intranasal inoculation and also that the peritoneal route would make it easier for infectious agents to reach the adventitia, which may be the main entrance for infectious agents. The ultrastructural study, which was performed only in one case for group, confirmed the presence of mycoplasma cells in the plaque, and of CP elementary bodies in the myocardial fibers, as well as of mycoplasma in the myocardial extracellular matrix. These data suggested that the studied infectious agents reached the circulation and many organs. The aggravation of atherosclerosis is probably caused by elements derived from infectious agents such as heat shock proteins or lipoproteins and not by direct presence of these agents in the lesion [22]. The mice fed with cholesterol enriched diet since the age of 8 weeks were infected at the age of 32 weeks and sacrificed at 40 weeks of age. This quite late period for inoculation increases the possibility that bacteria may be present in the atheroma plaques only as innocent bystanders, as they get a good breeding ground. However herein, the experimentally infected mice groups showed increased severity and different morphologies in atherosclerotic plaques than non infected animals. The presented results strongly point to that the studied infectious agents have a relevant role in atherosclerosis aggravation inducing injury directly by their presence in the plaques and/or indirectly by immune system activation.

All the infected groups showed low titers of serum antibodies to CP and MP. This is an expected result, since chlamydia and mycoplasma infections usually do not progress with high levels of antibodies probably due to the microbe escape mechanisms from the immune response [23,24]. Due to the small amount of blood collected from each animal, an individual antibody serum analysis could not be performed. The atherosclerosis was correlated more with the cholesterol levels than the antibodies to CP [25,26]. For this reason, the lack of individual animal antibody titers to CP or MP may be not so relevant for the interpretation of the studied infection.

The progression of atherosclerosis may be influenced by repeated microbe infections. Periods of increase and decrease of atherosclerotic lesions are seen by angiographic studies [27,28]. Bacterial lipopolysaccharides and endotoxins, autoimmunity due to molecular mimetization between the infectious agents, endovascular proteins such as Heat Shock Proteins and the activation of toll-like receptors by lipoproteins of the infectious agents are some of the mechanisms attributed to the development of inflammation and endothelial dysfunction in atherogenesis [29,30]. During both chronic and acute infections in humans, HDL levels decrease and may aggravate atherosclerosis [30], since this lipoprotein is important in reverse cholesterol transport and in the removal of oxidization products and inflammatory mediators from the circulation. However, in apoE KO mice, the loss of the ligand for lipid particle receptors is associated with an increase in total cholesterol due to mainly LDL particle accumulation. Basal cholesterolemia of apoE KO mice is up to five times higher than that of animals of the same strain without the genetic defect, that aggravate with cholesterol enriched diet [31]. Development of atherosclerotic lesions is also affected by cholesterol reverse transport in which apoE plays a pivotal role. In our study, lower level of LDL was seen in infected groups, mainly in MP group. However, the statistical analysis was not performed because we analyzed a pool of sera from each group.

Plaque rupture is not usually present in experimental atherosclerosis in animals including the apoE KO mice, which are considered an adequate experimental model for atherosclerosis studies [32]. In the present study it was not found ruptured plaques either. In humans, vulnerable plaques exhibited a third class of microbes, the Archaea [33], in close association with CP and MP.

## Conclusion

Intraperitoneal inoculation of *Chlamydia pneumoniae *(CP), *Mycoplasma pneumoniae *(MP) or both microbes caused aggravation of experimental atherosclerosis induced by cholesterol-enriched diet, with different characteristics. MP or CP caused more extensive atherosclerotic lesions in the aorta, CP resulted in increased plaque height with positive vessel remodeling and co-inoculation of MP + CP led to the development of more obstructive lesions due to smaller plaques associated with no vessel remodeling.

## Methods

### Animals

This study was approved by the Institutional Animal Welfare and Use Committee (Authorization number: SDS 2371/03/165). Animals were treated in accordance with the Guide for the Care and Use of Laboratory Animals [34].

Colonies of C57BL/6 apoE KO mice were obtained from original animals of Jackson Laboratories (Bar Harbor, ME). The foundation colonies were maintained in a Trexler isolator (Veco do Brasil, Campinas). Pups weaned at 21-days of age were housed in microisolator cages, under biosafety level 2 conditions, with free access to sterile water and regular irradiated rations. The mice were serologically negative for murine cytomegalovirus (MCMV), mouse hepatitis virus (MHV), minute virus of mice (MVM), *M. pulmonis*, *M. pneumoniae *and *C. pneumoniae*.

The mice were inoculated intraperitoneally with either 1 × 10^6 ^inclusion-forming units (IFU) of *C. pneumoniae *(CP), AR-39 (ATCC 53592), kindly provided by Prof. Mário Hirata of the Institute of Pharmaceutical Sciences of Sao Paulo University, and/or 1 × 10^6 ^colony forming units (CFU) of *M. pneumoniae *(MP) strain FH, (ATTC 15531), from the Institute of Biomedical Sciences of Sao Paulo University. Saline was the vehicle used to prepare the bacterial inoculum. The control group of non-infected mice were inoculated only with 100 μL of saline per mouse.

ApoE KO male mice aged 8-weeks were fed 1%-cholesterol (Sigma - C8503)-enriched diet for 24 weeks. After this period they were subdivided into four groups: a) ***Group CP ***(n = 9) inoculated with CP; b) ***Group MP ***(n = 13) inoculated with MP; c) ***Group CP+MP ***(n = 7) inoculated with CP and MP and d) ***Sham ***(n = 7) inoculated with saline. The infected animals were re-inoculated 4 weeks later, and sacrificed after 4 weeks, at 40 weeks of age.

At the end of the experiment, the mice were sedated with Ketamin (Parke-Davis) 25 mg/kg and Xylazin (Bayer) 5 mg/kg. An intracardiac puncture into the base of the left ventricle was performed with a 25-gauge, 3/4" needle to withdraw 1 ml of blood. The aorta was then fixed by perfusion for 3 to 5 min of 10% buffered formalin under physiological pressure. Two ascending aorta and one aortic arch segments, avoiding the regions of artery branch origin, were represented by three transversal rings, processed to be embedded in a single paraffin block, which was sliced in 5 μm serial sections and stained with Hematoxylin and Eosin and Masson's trichrome techniques.

A pool of sera from all animals in each group was obtained and stored at -20°C. The levels of total cholesterol and fractions were measured using an enzyme-based, colorimetric kit (Celm, Sao Paulo, SP- Brazil). For both CP and MP serum antibody quantitation, sera were pooled and titrated by serial, 2-fold dilution. CP AR-39 strain, acquired from the American Type Culture Collection (Manassas- VA, USA) was cultured in a Hep-2 lineage cells (Virology Section of the Adolfo Lutz Institute, Sao Paulo SP- Brazil). Wells containing the Hep-2 cells with CP inclusions were used to evaluate the antibody titers against CP by an in-house indirect immunofluorescence test, with fluorescein isothiocyanate-conjugated goat anti-mouse IgG (Sigma, St. Louis, USA). MP antibodies were detected by enzymatic inhibition as described elsewhere [35].

### Electron microscopy

One aorta fragment sectioned parallel to the first cross-section and one myocardial fragment nearby the aorta of the MP and CP + MP groups were sampled for electron microscopic examination, fixed in 3% glutaraldehyde and processed to be embedded in Araldite resin [36]. Thin sections were observed in a Philips EM-301 transmission microscope (Eindhoven, Netherlands) looking for MP cells and CP bodies in order to certify that the infection had occurred. The ultrastructural study was performed only in one case for group since it was not correlated with the amount of infectious agent bodies in the plaque with the aggravation of atherosclerosis, but only to verify whether the inoculated microbes had entered the circulation and reached the heart and artery walls.

### Microscopic evaluation of atherosclerosis

#### Semi-quantitative analysis

A semi-quantitative analysis was performed in all three aorta sections in the H&E slide by two independent observers, and a mean value was obtained using the following criteria: a) intimal and adventitial inflammation: 0 = absence, 1 = scarce lymphocytes, 2 = scarce foci and 3 = multiple or large foci of mononuclear infiltrate; b) Presence of hemorrhage, rupture or calcification of the plaque.

### Quantitative analysis

The quantitative analysis was performed measuring the most obstructive sample from the 3 sections in each case, and for the extension of atherosclerosis all plaques from the 3 sections were measured, as exemplified in Figure 2. The most severely obstructed vessel segment was measured based on the knowledge that the shortest diameter even in an oblique section of a tube is the same as the diameter in a cross-section at right angles to the longitudinal axis, fact that is referred to be valid also for wall thickness and luminal vessel diameter [37]. Figure 2A shows perpendicular measures, corresponding to the most obstructive section. A flat shape of the lumen suggests that the segment is collapsed despite of perfusion fixation. Collapsing might have happened during the embedding procedure. The three segments of each case measured around 6 mm length with less than 1 mm thickness. They were embedded in a same paraffin block and it was difficult to maintain them in perpendicular position. Therefore during embedding in a same paraffin block it was difficult to maintain them in perpendicular position and the sections look oblique. An irregular morphology suggesting a bifurcation area as exemplified in section 3, is probably caused by a positive versus absent or negative vessel remodeling induced by atherosclerosis development [38,39]. In this section, the upper side represents a fat plaque in both sides of the vessel, which is associated with a positive vessel remodeling, and the inferior part, a fibrotic plaque with no vessel remodeling.

The obstruction was evaluated by perpendicular measures to the vessel long axis, obtaining external diameter, plaque height, luminal diameter, % luminal obstruction and % fat area in the plaque. The measurements were made only in one plane, across the lowest diameter, in the Masson's Trichrome and H&E slides, using the Leica - Quantimet 500 Image Analysis System (Cambridge, UK), by obtaining the following variables: a) vessel diameter (distance comprised by the external elastic membrane); b) potential luminal diameter (distance comprised by the internal elastic membrane); c) height of the plaque, and d) luminal diameter. The % luminal obstruction was calculated using the formula: (potential luminal diameter - luminal diameter)/potential luminal diameter × 100). The % lipid content was calculated by measuring the non-stained plaque regions (total plaque area less the fibromuscular area detected by automatic color detection).

#### Plaque area/internal surface

The extension of atherosclerosis was evaluated measuring the total intimal area present in the three sections of the case divided by the internal elastic membrane perimeter (mm^2^/mm) ratio, as shown in Figures 2B and 2C.

### Statistical analysis

Analysis of variance, Bonforroni and Dunn's tests were used, with significance at P ≤ 0.05 (ANOVA test and Dunn's for non-normally distributed values or Bonferroni's test for normally distributed values). T test was used when significance was not reached with ANOVA test in order to point possible differences if only two groups were compared.

## Authors' contributions

SBD and MLH - carried out the molecular genetic studies, participated in the sequence alignment and drafted the manuscript. MMR and MLH - participated in the design of the study and performed the statistical analysis. JTO; SAPP; RNI and LFPF - participated in the sequence alignment. MHL - conceived of the study, and participated in its design and coordination and helped to draft the manuscript. JT and FCP - carried out the immunoassays. All authors read and approved the final manuscript.
